# Efficacy of Semaglutide in Treating Obesity: A Systematic Review of Randomized Controlled Trials (RCTs)

**DOI:** 10.7759/cureus.32610

**Published:** 2022-12-16

**Authors:** Mahvish Anam, Shrinkhala Maharjan, Zainab Amjad, Abdelrahman Abaza, Advait M Vasavada, Akhil Sadhu, Carla Valencia, Hameeda Fatima, Ijeoma Nwankwo

**Affiliations:** 1 Internal Medicine, California Institute of Behavioral Neurosciences & Psychology, Fairfield, USA; 2 Pathology, California Institute of Behavioral Neurosciences & Psychology, Fairfield, USA; 3 Family Medicine, California Institute of Behavioral Neurosciences & Psychology, Fairfield, USA

**Keywords:** systematic review, glp-1 receptor agonists, safety, efficacy, obesity treatment, semaglutide, glucagon like peptides

## Abstract

Obesity is a major health problem worldwide resulting in numerous health conditions such as heart disease, stroke, type 2 diabetes (T2D), and certain types of cancer which are among the leading causes of premature preventable deaths. Recently, glucagon like peptide-1 receptor agonists (GLP-1 RA) has been identified as the most promising intervention in treating obesity. Our systematic review aims to analyze the efficacy of semaglutide, a GLP-1RA in treating obesity. We searched PubMed, Science Direct, and Google Scholar databases to review and distill full-text articles based on the eligibility criteria and involved 12 papers of clinical trials. The review found that semaglutide is safe and effective in treating obesity, and complications reported were primarily gastrointestinal events. Further exploration with more number of clinical trials involving greater sample size and lengthier time of follow-up is essential to determine its efficacy and safety in a diverse group of individuals who are overweight or obese and the dose required along with the duration of treatment.

## Introduction and background

**Figure 1:**
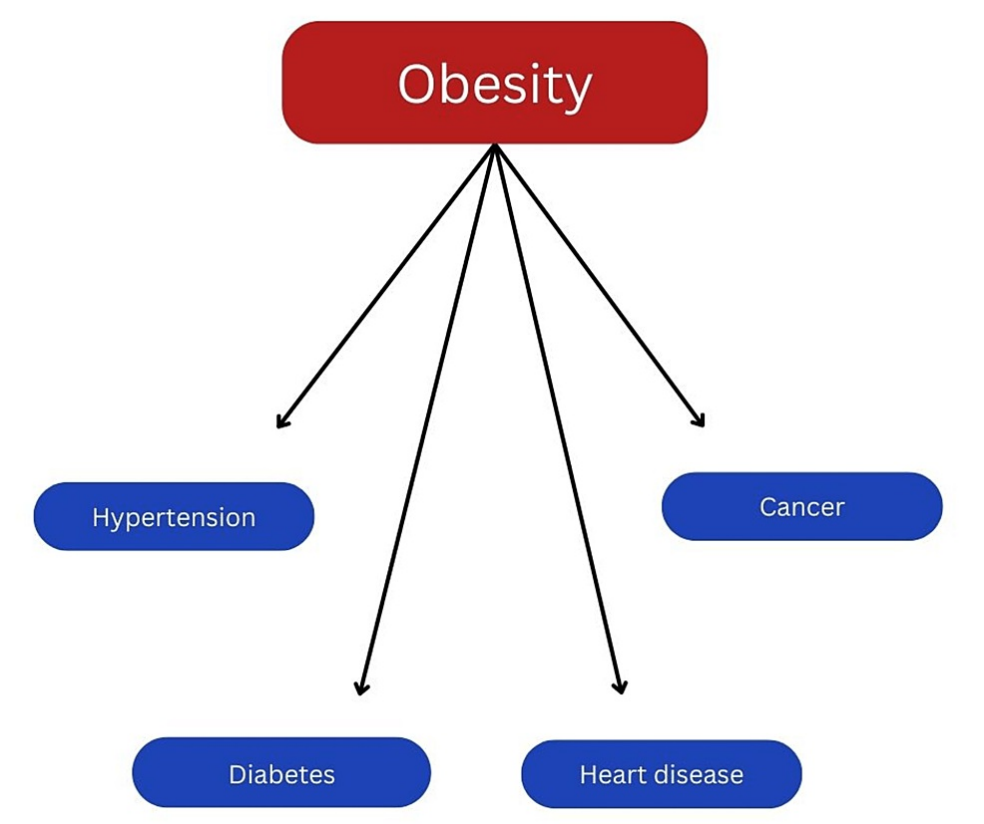
Obesity leading to various health conditions. Original Image: Advait Vasavada

Obesity, a multifactorial disease, is a leading cause for increased incidence of cardiovascular risk factors, including dyslipidemia, type 2 diabetes (T2D), hypertension, and sleep disorders [1]. The higher the body mass index (BMI), the greater is the risk of morbidity and mortality [2]. Reduction in BMI decreases the risk of development of type 2 diabetes mellitus, hypertension, acanthosis nigricans, and depression to name a few [3-6]. Being a global epidemic, the prevalence of obesity in the United States has increased from 30.5% in 1999-2000 to 41.9% in 2017-2020 [7], with an estimated annual burden of nearly $173 billion dollars in 2019 [8]. According to CDC, overweight and obesity are defined as BMI more than 25 and 30, respectively [9]. Hence, obesity leads to a myriad of other diseases (Figure 1).

Lifestyle interventions and dietary modifications are the initial approaches to weight reduction [10]. Currently, Food and Drug Administration (FDA) has approved five drugs for long-term use for obesity which includes orlistat, phentermine-topiramate, naltrexone-bupropion, liraglutide, and semaglutide of which phentermine is the commonly prescribed option [11]. Semaglutide 2.4 mg is to be administered subcutaneously, once a week for adults with overweight (body mass index >27 kg/m^2^) with at least one weight-associated condition [for instance, high blood pressure, type 2 diabetes (T2DM), or high cholesterol], or adults with BMI of 30 kg/m^2^ or greater, received FDA approval in 2021 [11].

 Secreted from the L‐cells in the small intestine, glucagon‐like peptide (GLP)‐1, an incretin hormone lowers blood glucose levels by stimulating insulin and inhibiting glucagon secretions in a glucose level dependent way from the pancreatic islets [12]. A decrease in appetite and craving for food, a relatively low proclivity for fatty, energy‐rich foods, and better control of eating are the most likely mechanisms for semaglutide-induced weight loss [13]. This systematic review has the primary objective to study the use of semaglutide for weight loss.

## Review

Study design

The Preferred Reporting Items for Systematic Review and Meta-Analyses (PRISMA) 2020 Guidelines [14-15] are standard for systematic reviews and were used to conduct and record the data presented in this systematic review. 

Sources of data collection

Three databases were used to collect and review relevant articles: PubMed, ScienceDirect, and Google Scholar. Each database was properly screened using the keywords: glucagon-like peptides, semaglutide, obesity treatment, efficacy, and safety.

Search strategy 

The use of Medical Subject Headings (MeSH) was advocated to filter the search strategy on PubMed further. The eventual search strategy was formulated as follows: ("glucagon like peptides/administration and dosage"[MeSH Terms] OR "glucagon like peptides/therapeutic use"[MeSH Terms]) AND "obesity/drug therapy"[MeSH Terms]. For other databases, keywords were used to get the relevant articles.

Inclusion and exclusion criteria

We included, selected, evaluated, and identified articles published in the English language. Randomized clinical trials, adult population, human-based studies, obtainability of free full text, and articles published between 2012 and May 2022. Studies in a non-English language, animal/preclinical studies, review articles, and non-full-text articles were excluded.

Data extraction

The relevant studies were screened, distilled, and collected by two independent researchers, anonymously through the Rayyan Software (Rayyan Systems Inc., Cambridge, Massachusetts) [16]. The intervention and outcome were carefully and closely monitored and extracted. The data extracted from the studies were classified according to the author, year of publication, study type/design, results, and conclusion. Screening of abstracts and titles was done initially using the Rayyan software. Then, two authors (Mahvish and Shrinkhala) assessed the data independently and filtered for all studies that were identified. A total of 1,000 articles were identified after applying the criteria for the search. A number of results from each database were recorded.

Out of the 1000 publications, 253 duplicates were found and 747 articles remained after eliminating duplicates. A total of 747 articles were screened based on the inclusion and exclusion criteria, and 12 papers were stipulated after the screening process. 

Risk and quality assessment

We included 12 randomized controlled trials (RCTs) in our study. Each study included in the systematic review evaluated the risk and quality assessment. The Revised Cochrane’s Risk of Bias Tool was utilized for RCTs. The trials satisfying the criteria of > 70% for quality and grade were hand-picked for the systematic review. 

Results

**Figure 2:**
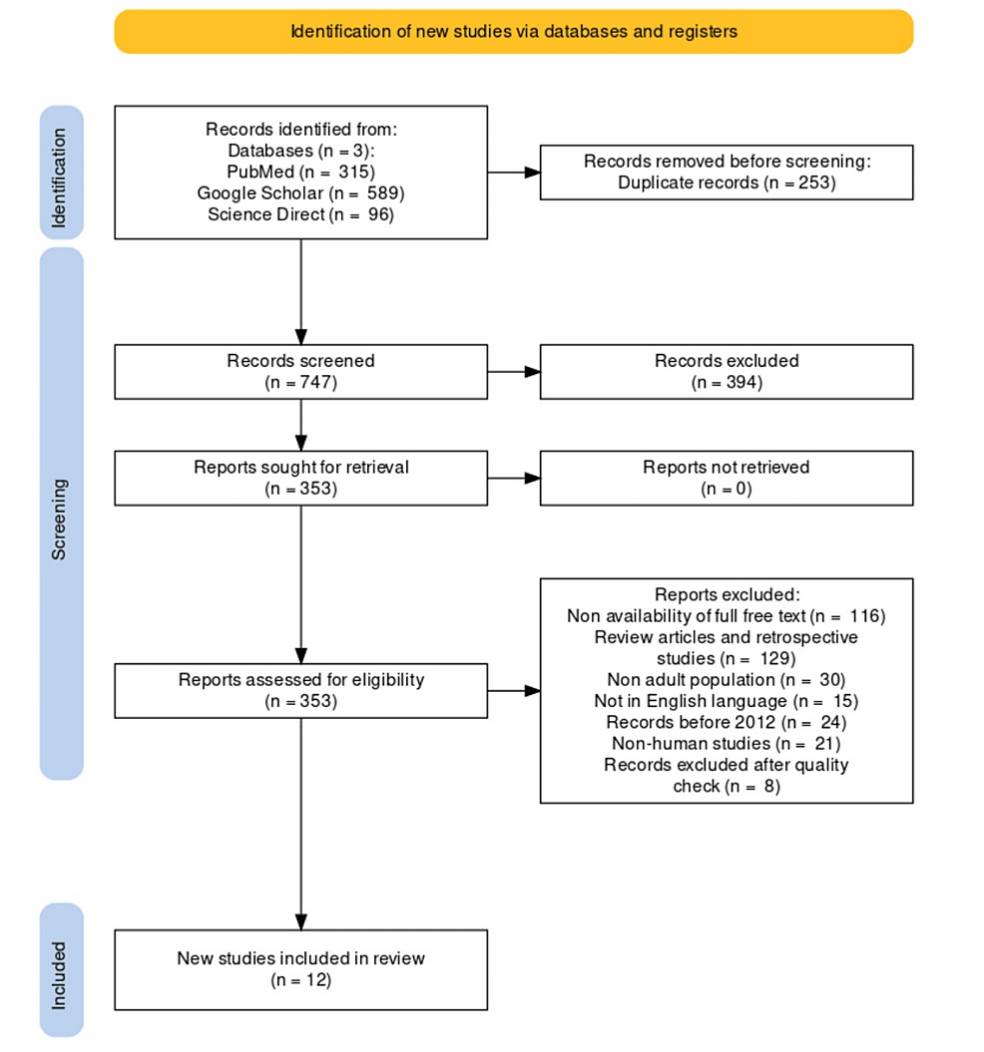
PRISMA flow diagram for included articles. PRISMA, Preferred Reporting Items for Systematic Review and Meta-Analyses

A total of 1000 articles were selected using the above search strategy criteria listed in the methods section. The articles were screened based on the title and abstract related to semaglutide and obesity treatment. After a detailed assessment, we applied inclusion and exclusion criteria and included 12 studies, and a complete PRISMA flow diagram [17] was created (Figure 2).

Risk of bias assessment

**Figure 3:**
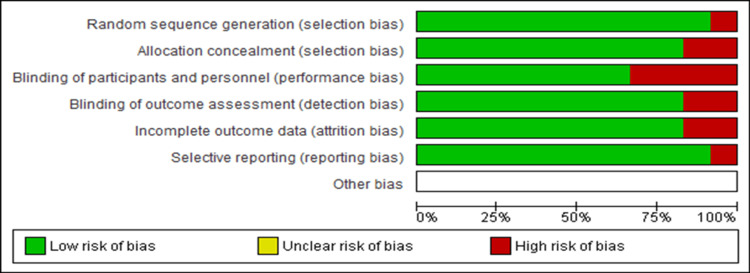
Graph showing the risk of bias of 12 included articles.

We used 12 randomized clinical trials (RCTs) in our study and assessed the risk of bias using the Cochrane Risk of Bias tool (Figure 3).

**Figure 4:**
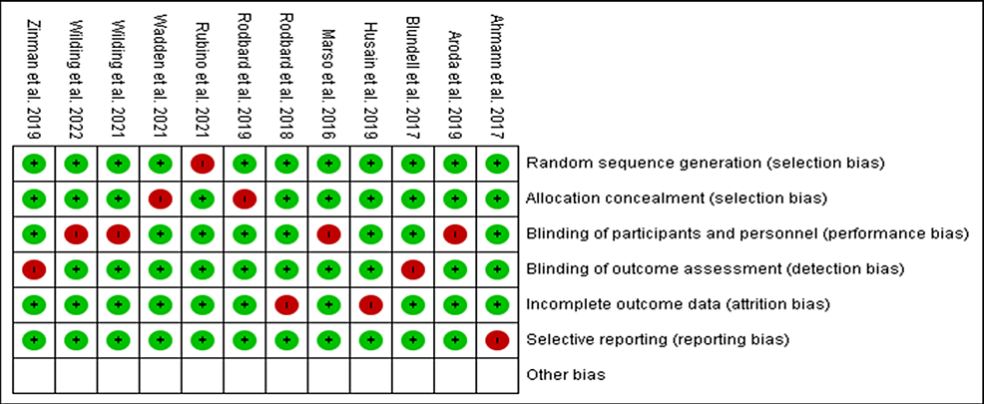
Summary of the risk of bias assessment.

According to the Cochrane risk of bias evaluation, the majority of the included research obtained high ratings and was classified as having a low risk of bias for nearly all of the evaluation categories. Red spots imply greater bias vulnerability, green areas suggest moderate risks, and unshaded areas show uncertain risks (Figure 4). Articles with high-risk components were ignored because their findings might have changed and led to conflicts in this article's conclusions.

Discussion

The participant details, a dose of semaglutide, and major outcomes are delineated in the table below (Table 1).

**Table 1:** Study characteristics. RCT, randomized controlled trial

Study ID	Study design	Participants			Intervention/Placebo	Follow-up period (weeks)	Main outcomes/efficacy
Author (year)		No	Mean age (year)	Gender (n) males/females	Semaglutide vs the same dose of placebo (dose volume administered)		Mean weight reduced after semaglutide administered (kg)
Blundell et al. [13]	RCT	30	42	20/ 10	0.25 mg/ 0.5 mg/ 1 mg/week	12	5
Aroda et al. [18]	RCT	703	55	358/345	3 mg/ 7 mg/ 14 mg/week	26	4.1
Rodbard et al. [19]	RCT	787	58	397/390	14 mg vs 25 mg/week	26	3.8
Husain et al. [20]	RCT	3183	66	2176/1007	14 mg/week	64	4.2
Zinman et al. [21]	RCT	731	61	397/334	7 mg/ 14 mg/ 3 mg/week	52	3.7
Ahmann et al. [22]	RCT	813	56.4	447/366	0.25 mg/ 0.5 mg/ 1 mg vs 2 mg/week	56	5.6
Rodbard et al. [23]	RCT	396	59	222/174	0.5 mg/ 1 mg/week	30	6.4
Marso et al. [24]	RCT	3297	64	2002/1295	0.5 mg/1 mg/week	104	4.9
Wilding et al. [25]	RCT	1961	46	508/1453	2.4 mg/week	68	15.3
Wadden et al. [26]	RCT	611	46	116/495	2.4 mg/week	68	16.9
Rubino et al. [27]	RCT	803	46	169/634	2.4 mg/week	68	11.1
Wilding et al. [28]	RCT	327	49	107/220	2.4 mg/week	120	18.25

Mechanism of weight loss

Blundell et al. conducted randomized, double‐blind, placebo‐controlled, two‐period crossover trials, in 30 subjects with obesity to study the effects of 12 weeks of treatment with the therapy of once‐weekly subcutaneous semaglutide [13]. Subjects were randomly assigned to one of two treatment groups: semaglutide-placebo or placebo-semaglutide and it included two 12‐week crossover treatment periods, separated by a wash‐out period of five to seven weeks [13]. Semaglutide‐induced weight loss was associated with a relatively higher reduction of body fat than lean body mass, decreased energy intake due to a decrease in appetite, better control of eating, fewer food cravings, and a lower relative preference for fatty, energy‐dense foods and not the result of increased energy expenditure [13].

Pioneer trials

Pioneer 1 Trial, an RCT conducted by Aroda et al. to compare the efficacy and safety of oral semaglutide to placebo in patients with type 2 diabetes which included 703 patients for 26 weeks who were randomized to receive 3, 7, or 14 mg oral semaglutide or placebo [18]. At the end of 26 weeks, patients with higher doses (7 mg, 14 mg) achieved statistically significant reductions in body weight compared to placebo [18].

Rodbard et al. conducted Pioneer 2 Trial, to compare the efficacy and safety of oral semaglutide to empagliflozin in type 2 diabetes mellitus (T2DM) uncontrolled with metformin alone [19]. In the trial, 412 patients were randomized to receive once-daily oral semaglutide 14 mg and 410 patients received empagliflozin 25 mg for 52 weeks [19]. In addition to reduction in glycated hemoglobin (HbA1c), body weight reduction was seen with both treatments, however, the superiority of oral semglutide was not seen at week 26 [19]. Significantly greater weight losses were noticed at 52 weeks in oral semaglutide group compared to empagliflozin where stability in weight loss was noted after 26 weeks through 52 weeks [19].

In a similar study, Pioneer 6 conducted by Husain et al. to study the cardiovascular safety of oral semaglutide 14 mg showed there was -4.2 kg change in body weight from baseline in semagltuide group vs -0.8 kg in the placebo group [20]. The Pioneer 6 trial ran for 15.9 months with 3183 patients, was a randomized, double-blind, placebo-controlled trial [20].

The efficacy, safety, and tolerability of oral semaglutide added to insulin with or without metformin was studied in Pioneer 8 Trial, by Zinman et al., where T2DM patients were randomized to oral semaglutide 3 mg (N = 184), 7 mg (N = 182), or 14 mg (N = 181) or to placebo (N = 184) for 52 weeks [21]. From baseline to week 26, the estimated mean body weight changes were -1.4, -2.4, -3.7, and -0.4 kg for oral semaglutide 3, 7, and 14 mg and placebo, respectively [21]. This study provided statistically significant reduction of >5% body weight in contrast to placebo over 52 weeks in patients with type 2 diabetes poorly controlled with insulin with/without metformin. Furthermore, this result was observed in a dose-dependent fashion [21].

Sustain trials

Sustain 3 trial conducted for 56 weeks included once-weekly semaglutide 1.0 mg subcutaneous or once-weekly exenatide extended release (ER) 2.0 mg subcutaneous injections resulted in a mean reduction of 5.6 kg body weight over the one-year period which was almost three times larger than exenatide ER [22]. In addition, a weight loss response of ≥5% was seen in 52% receiving semaglutide compared to 17% of subjects receiving exenatide ER [22].

When added to basal insulin, subcutaneous semaglutide in patients with uncontrolled T2DM it remarkably reduced HbA1c and body weight when compared to placebo in Sustain 5 Trial, a double-blinded RCT [23]. In the study participants receiving semaglutide 0.5 mg, 1 mg and placebo, >5% reduction of body weight was noted in 42%, 66%, and 11% respectively at the end of 30 weeks [23]. 

Sustain 6 trial conducted by Marso et al. at 230 sites in 20 countries assigned 3297 patients with type 2 diabetes who were on a standard-care regimen to receive once-weekly subcutaneous semaglutide (0.5 mg or 1.0 mg) or placebo for 104 weeks [24]. Mean body weight in the semaglutide group, as compared with the placebo group, was 2.9 kg lower in the group receiving 0.5 mg and 4.3 kg lower in the group receiving 1.0 mg [24].

Step trials

Step 1 trial was a randomized, double-blind, placebo-controlled, conducted by Wilding et al. and it included 1961 nondiabetic adult participants with obesity or overweight [25]. Participants were randomly assigned in a 2:1 ratio, to receive either semaglutide 2.4 mg subcutaneously once a week for 68 weeks or placebo, in addition to lifestyle intervention [24]. The estimated end results at 68 weeks were mean change in body weight of −14.9% with 2.4 mg semaglutide, as compared with −2.4% with placebo [25]. The threshold of losing more than 5%, 10%, and 15% body weight were reached by 86.4%, 69.1%, and 50.5% participants in semaglutide group when compared to 31.5%, 12.0%, and 4.9% participants in the placebo group [25].

Like Step 1 trial, Wadden et al. conducted Step 3 trial, a randomized, double-blind study, consisting of 68 weeks where participants were randomly assigned (2:1) to either subcutaneous semaglutide, 2.4 mg or placebo, along with a low-calorie diet for the first 8 weeks and intensive behavioral therapy [26]. The estimated mean body weight change from baseline was -16.0% for semaglutide compared to -5.7% for placebo at the end of 68 weeks and there was an additional 3%-5% reduction in body weight when used in adjunct to dietary modifications [26].

The above-mentioned studies concluded that semaglutide significantly lowers body weight and is efficient in treating obesity in the non-diabetic or diabetic population.

Effects of continued 2.4 mg SC semaglutide

Rubino et al. conducted Step 4 trial, where 803 participants after achieving a mean weight loss of 10.6% with weekly subcutaneous semaglutide, 2.4 mg, were randomly allotted to continue SC semaglutide or switch to the placebo group [27]. Some 40% of participants who continued semaglutide lost an additional 10% of body weight during the randomized period with a mean change in weight of −7.9% vs +6.9% in semaglutide and placebo groups respectively from week 20 to week 68 [27].

Weight regain after withdrawal of treatment

Step 1 trial extension conducted by Wilding et al. included 327 participants. From week 0 to week 68, mean weight loss was 17.3% with semaglutide and 2.0% with placebo [28]. Following treatment withdrawal, semaglutide and placebo participants regained 11.6 and 1.9 percentage points of lost weight, respectively, by week 120, resulting in net losses of 5.6% and 0.1% respectively [28].

Adverse effects

In Pioneer trials, nausea of mild to moderate intensity was noted which was the main reason for premature discontinuation [13, 19-21]. The mean pulse rate increased significantly with oral semaglutide 14 mg but not with 3 or 7 mg in Pioneer 1 Trial as well as 2-4 beats increase in pulse rate in the pioneer 8 trial [13, 20]. No clinically relevant changes in blood pressure or other safety laboratory assessments were noticed [13, 19-21]. Diabetic retinopathy-related adverse events were also reported [13, 19-21]. And malignant neoplasms were identified in 1.7% of patients in the oral semaglutide group and 0.5% in the empagliflozin group in the Pioneer 2 trial [19].

Sustain trials reported gastrointestinal adverse effects mainly as a reason for discontinuation of the drug [22-24]. There were also reported cases of diabetic retinopathy and an increase in mean pulse rate of around 2 beats per minute [22-24]. There was a confirmed case of metastatic pancreatic cancer with an onset date of 65 days after the end of treatment in Sustain 5 Trial, in addition to one more case in Sustain 6 trial [23-24]. Nine subjects confirmed acute pancreatitis in Sustain 6 trial [25].

In Step 1 trial, one gallbladder-related adverse event mostly cholelithiasis was reported [24]. Some 4.9% of subjects in Step 3 trial reported cholelithiasis in addition to three subjects reporting basal cell carcinoma, breast cancer, and papillary thyroid cancer each [26].

From our analysis, we found that semaglutide causes few adverse events mostly related to gastrointestinal disorders, an increase in the pulse rate, and diabetic retinopathy. Nausea is the most frequent and the most important factor for the discontinuation of drugs. No serious events such as cardiovascular events were reported.

Limitations

The systematic review has certain limitations. Firstly, a relatively small number of studies were included in the review. Secondly, only articles available in the English language and articles only available for free were screened. Lastly, the overweight or obese individuals' sample size was relatively small. 

## Conclusions

We assessed the use of oral and subcutaneous semaglutide in our article. Most of the studies included demonstrated a positive effect of semaglutide for obesity treatment. Some studies also revealed a dose-dependent effect of the therapy in those with poorly controlled diabetes. Hence, the result seems to be overwhelmingly in favor of the use of the drug. There were a few adverse events noted mostly significant for gastrointestinal (GI)-related disorders, which led to premature discontinuation of the drug. Our review enforces the need for more trials with longer duration, large study groups, and a follow-up period after withdrawal is needed. Also, meta-analytical studies are recommended to quantitatively assess its use.

## References

[1] Powell-Wiley TM, Poirier P, Burke LE (2021). Obesity and cardiovascular disease: a scientific statement from the American Heart Association. Circulation.

[2] Jensen MD, Ryan DH, Apovian CM (2014). 2013 AHA/ACC/TOS guideline for the management of overweight and obesity in adults: a report of the American College of Cardiology/American Heart Association Task Force on Practice Guidelines and The Obesity Society. Circulation.

[3] Knowler WC, Barrett-Connor E, Fowler SE, Hamman RF, Lachin JM, Walker EA, Nathan DM (2002). Reduction in the incidence of type 2 diabetes with lifestyle intervention or metformin. N Engl J Med.

[4] Siebenhofer A, Jeitler K, Horvath K, Berghold A, Posch N, Meschik J, Semlitsch T (2016). Long-term effects of weight-reducing drugs in people with hypertension. Cochr Datab Syst Rev.

[5] Maitra SK, Rowland Payne CM (2004). The obesity syndrome and acanthosis nigricans. Acanthosis nigricans is a common cosmetic problem providing epidemiological clues to the obesity syndrome, the insulin-resistance syndrome, the thrifty metabolism, dyslipidaemia, hypertension and diabetes mellitus type II. J Cosmet Dermatol.

[6] Dixon JB, Dixon ME, O'Brien PE (2003). Depression in association with severe obesity: changes with weight loss. Arch Intern Med.

[7] (2022). National Health and Nutrition Examination Survey 2017-March 2020 Prepandemic Data Files Development of Files and Prevalence Estimates for Selected Health Outcomes. Vital Health Stat and series.2021.

[8] Ward ZJ, Bleich SN, Long MW, Gortmaker SL (2021). Association of body mass index with health care expenditures in the United States by age and sex. PLoS One.

[9] (2022). Defining adult overweight & obesity. https://www.cdc.gov/obesity/basics/adult-defining.html.

[10] Isaacs D, Prasad-Reddy L, Srivastava SB (2016). Role of glucagon-like peptide 1 receptor agonists in management of obesity. Am J Health Syst Pharm.

[11] Idrees Z, Cancarevic I, Huang L (2022). FDA-approved pharmacotherapy for weight loss over the last decade. Cureus.

[12] Kim W, Egan JM (2008). The role of incretins in glucose homeostasis and diabetes treatment. Pharmacol Rev.

[13] Blundell J, Finlayson G, Axelsen M, Flint A, Gibbons C, Kvist T, Hjerpsted JB (2017). Effects of once-weekly semaglutide on appetite, energy intake, control of eating, food preference and body weight in subjects with obesity. Diabetes Obes Metab.

[14] Page MJ, Moher D, Bossuyt PM (2021). PRISMA 2020 explanation and elaboration: updated guidance and exemplars for reporting systematic reviews. BMJ.

[15] (2022). Rayyan - a web and mobile app for systematic reviews. https://www.rayyan.ai/.

[16] Khan AA, Mannan V, Pervaiz MA (2022). The role of glucosamine and chondroitin sulfate in the prevention of colorectal cancer: a systematic review. Cureus.

[17] Haddaway NR, Page Page, MJ MJ, Pritchard CC, McGuinness LA (2022). PRISMA2020: an R package and Shiny app for producing PRISMA 2020-compliant flow diagrams, with interactivity for optimised digital transparency and Open Synthesis Campbell Systematic Reviews, 18, e1230. Campbell Systematic Reviews.

[18] Aroda VR, Rosenstock J, Terauchi Y (2019). PIONEER 1: randomized clinical trial of the efficacy and safety of oral semaglutide monotherapy in comparison with placebo in patients with type 2 diabetes. Diabetes Care.

[19] Rodbard HW, Rosenstock J, Canani LH (2019). Oral semaglutide versus empagliflozin in patients with type 2 diabetes uncontrolled on metformin: the PIONEER 2 trial. Diabetes Care.

[20] Husain M, Birkenfeld AL, Donsmark M (2019). Oral semaglutide and cardiovascular outcomes in patients with type 2 diabetes. N Engl J Med.

[21] Zinman B, Aroda VR, Buse JB (2019). Efficacy, safety, and tolerability of oral semaglutide versus placebo added to insulin with or without metformin in patients with type 2 diabetes: the PIONEER 8 trial. Diabetes Care.

[22] Ahmann AJ, Capehorn M, Charpentier G (2018). Efficacy and safety of once-weekly semaglutide versus exenatide ER in subjects with type 2 diabetes (SUSTAIN 3): a 56-week, open-label, randomized clinical trial. Diabetes Care.

[23] Rodbard HW, Lingvay I, Reed J (2018). Semaglutide added to basal insulin in type 2 diabetes (SUSTAIN 5): a randomized, controlled trial. J Clin Endocrinol Metab.

[24] Marso SP, Bain SC, Consoli A (2016). Semaglutide and cardiovascular outcomes in patients with type 2 diabetes. N Engl J Med.

[25] Wilding JP, Batterham RL, Calanna S (2021). Once-weekly semaglutide in adults with overweight or obesity. N Engl J Med.

[26] Wadden TA, Bailey TS, Billings LK (2021). Effect of subcutaneous semaglutide vs placebo as an adjunct to intensive behavioral therapy on body weight in adults with overweight or obesity: the STEP 3 randomized clinical trial. JAMA.

[27] Rubino D, Abrahamsson N, Davies M (2021). Effect of continued weekly subcutaneous semaglutide vs placebo on weight loss maintenance in adults with overweight or obesity: the STEP 4 randomized clinical trial. JAMA.

[28] Wilding JP, Batterham RL, Davies M (2022). Weight regain and cardiometabolic effects after withdrawal of semaglutide: the STEP 1 trial extension. Diabetes Obes Metab.

